# The Mechanism of Respiration and Circulation, or an Explanation of the Nature of Congestion of the Lungs. A Physiological and Pathological Inquiry

**Published:** 1846-07

**Authors:** 


### Der Mechanismus der Respiration und Circulation, oder das explicirte Wesen der Lungenhyperämien. Eine Physiologisch-pathologische Untersuchung. Von Dr. *A. Mendelssohn*. Berlin 1845.

### The Mechanism of Respiration and Circulation, or an Explanation of the Nature of Congestion of the Lungs. A Physiological and Pathological Inquiry. By Dr. *A. Mendelssohn*.

The author, in the physiological division of his work, has two objects principally in view ; in the first place, to determine the cause of the well-known obstruction occurring in the pulmonary circulation in consequence of any impediment to the free admission of air into the lungs ; and, secondly, to show that a particular influence is exerted by the normal expansion of the lungs, on the movement of the blood within the pulmonary artery and its capillaries. It does not appear that Dr. Mendelssohn is acquainted with the numerous researches which have been made in this country in connection with the first part of his inquiry ; and the consequence is that, on the most important points of which he treats, the author has been anticipated, especially by the extended and valuable experimental inquiry of Dr. Reid.

---

In 1843, however, Dr. Pliny Earle stated, in the American Journal of Medical Science, that there were 20 public asylums, providing 2647 beds, and possessing 671 acres of ground.

There are no nerves to which so much attention has been directed as those of the par vagum, not only on account of their intrinsic importance, but more especially because physiologists have been anxious to avail themselves of the unwonted facilities these cords present for experimental investigation, to determine the influence which is exerted by the nervous system upon some of the most recondite operations of animal bodies—digestion, secretion, circulation, and the production of animal heat.

Although the erroneous theories which owed their origin mainly to the well-known experiments of Dr. W. Philip and Sir B. Brodie, have been gradually replaced by more accurate views, we are still desirous of offering a few remarks upon the functions of organic life and their relations to the nervous system, as upon these subjects many misconceptions still prevail.

One of the most valuable results which has in late years sprung from the comprehensive study of the organic laws, is the conviction that the various processes by which nourishment is accomplished in the animal, are essentially dependent on the same principles and are submitted to the same laws, as those which govern the phenomena of nutrition in the plant. The respiratory action, for instance, is really the same, whether it be accomplished by the leaf of a vegetable or by the lung of an animal; in both cases the immediate object is the introduction of oxygen into the system, and the consequent formation of carbonic acid; the ultimate end being the development of caloric. True it is the conditions for realising these results are very different in the two kingdoms; for, whereas, in the case of the plant, all that is required is the contact of the leaf with the atmosphere, and the circulation of the sap; in the case of the animal, a complicated series of actions is required for bringing about the contact of the blood with the atmospheric air. Now, if we enquire what share the nervous system has in the respiratory function, it would not be difficult to prove that it is merely concerned with these incidental actions—the movements, namely, of the chest and the circulation of the blood; for as to the ultimate actions, the introduction of oxygen, the evolution of carbonic acid, and the development of caloric, they are phenomena as strictly chemical in their nature as when they occur in the vegetable. Considerations of this kind will enable the physiologist to assign to the nervus vagus its real share in the breathing process, and thus to avoid the error of supposing that it has some obscure and unknown power of generating animal heat. A similar train of reasoning might be applied to the secerning function, which, as it goes on most actively in vegetables where there are no nerves, as it is effected in animals by the same mechanism as in plants, namely, cells, and as moreover, it continues after the nerves have been divided, we must presume is essentially independent of the nervous centres. But, as in the instance of respiration, it is very possible to conceive, that although the act of secretion is thus independent, the conditions necessary to bring it about, the circulation of the blood for example, may require the interposition of nervous agency; and moreover, which has not been sufficiently attended to in this inquiry, the discharge of fluids may, in many cases, as it certainly does in some, demand the influence of the nerves.

The experimental part of the work of Dr. Mendelssohn does not present, for the reason above stated, much novelty. He found, as Dr. Reid had already determined, that the division of *one* vagus nerve, produced no injurious effect upon the lung on the same side, nor upon the general health of the animal; in five experiments of this kind "the lungs were perfectly healthy," (p. 24). The effects of dividing the nerves on both sides were the same as those ascertained by preceding experimentalists. The fourth and fifth series of experiments are interesting, as showing that paralysis of the glottis, induced by dividing the recurrent laryngeal nerves, produces the same fatal effects in the lungs as if the two vagi nerves were themselves cut through; vascular congestion, increased density, so that pieces of the lungs sank in water, effusion of a reddish or yellowish matter containing nucleated cells, corpuscles, and in some parts the red particles of the blood, were the results. The number of respirations was diminished, and there was a rustling or grating sound; out of eight rabbits operated on, five died in from two to five days, but two recovered. (P. 29-36).

Although the author has thus shown that some of the effects of dividing the recurrent nerves are similar to those attending the section of the par-vagum, the vast difference and superiority of these latter nerves, as the excitors of the respiratory movements, a distinction apparently overlooked in the work before us, must never escape the notice of the physiologist in researches of this nature.

Some other experiments are related, but as the general views of the author may be gathered from the subjoined extracts, in which his principal conclusions are summed up, it is not necessary to detail them.

"The paralysis of the branches of the nervus vagus which are distributed to the lungs, is not the cause of the affection which develops itself in the lungs after the division of both nervi vagi in the neck.

"The excision of both nervi recurrentes causes the same affection in the lungs, though somewhat later, as the excision of the nervi vagi; from which it must be inferred that the paralysis of these two nerves, which ensues upon the excision of the two nervi vagi in the neck, is an essential cause of the above-mentioned affection.

"The conclusion of Longet, in his physiological resumé on the functions of the nervi vagi and recurrentes, that 'animals deprived of their recurrent nerves respire more quickly than in the normal state,' must rest upon false or misinterpreted facts, since my researches constantly prove the contrary.

"The preceding experiments and their results prove that, the different functions which have been attributed to the branches of the nervi vagus going to the lungs, are erroneous; they also shew that nothing more can be at present affirmed than that these branches bestow the sensation possessed by the mucous membrane of the bronchi, and that they contain motorial fibres for the bronchi.

"The phenomena resulting from the division of the nervi vagi or recurrentes, consist of a retardation of the circulation, a dilatation of the capillary vessels, an exudation of plasma into the tissue of the lungs and into the air-cells, and of the usual metamorphoses of this exudation: they are therefore in all respects analogous to the process, which, in pathology, is termed pneumonia.

"The stasis of the blood in the capillary vessels of the lungs, which is the cause of the alterations above described, is, as such, not yet known, and all that is seen is the alteration produced by the loss of the influence of the vagus upon the lungs and their function, whilst the cause of the change is not determined. I have, however, seen the same alteration that is caused by the excision of the nervi recurrentes, produced by opening the cavity of the pleura, by injecting a thin fluid into the bronchi, or by introducing a hard body into one of the bronchial tubes. If, out of all these researches, we select that which is common, the law will appear—that the hindrance to the entrance of the air during inspiration, or more accurately to the expansion of the air-cells, determines as a consequence a stagnation in the capillary vessels of the lungs.

"The preceding facts justify the presumption that the influence exerted by respiration upon the systemic circulation, which Poiseuille has determined, may be much farther extended than has heretofore been supposed; that, in fact, this influence, in virtue of a power which is generated in the lesser circulation by the expansion and contraction of the lungs, is communicated to the vessels running to and within the lungs; this presumption attains to certainty by experiments.

"The afflux or increased pressure of the venous blood towards the chest, and the diminished pressure of the arterial, is not determined, as hitherto supposed, by the rarefaction of the air within the thorax and the corresponding augmented atmospheric pressure, but is the consequence of the suction (*aspiration*) exerted on the blood by the elongation of the pulmonary artery, together with its branches and the capillary vessels of the air-cells.

"The suction, which is exercised by means of the elongation of the pulmonary vessels upon the blood contained in the veins, constitutes also the mechanism by which the processes of digestion and nutrition are connected with respiration, inasmuch as the chyle and the lymph are, by means of the ductus thoracicus opening into the subclavian vein, brought under the influence of this latter process: the materials of nutrition are, as it were, pumped into the blood by means of the expansion of the lungs.

"If from the starting-point which has now been gained, the results of these experiments be once more regarded, it is easy to show why, after the section of the nervi vagi, of the nervi recurrentes, after the introduction of a hard body into the bronchus, after an opening into the pleural sac, a stagnation must arise in the lesser circulation, and as a consequence in the greater circulation also; in other words, how in these instances pneumonia is caused, whilst after a narrowing of the air-tube, or after ligaturing the abdomen, only a stasis is determined in the greater circulation, whilst the lungs remain free.

"The influence above described, which the expansion and contraction of the lungs exert upon the greater circulation, is the cause of the closing of the fœtal passages, of the alteration of the circulation, and of the hypertrophy of the left heart developed in the first week of life."

These experiments and their results are so far useful as confirming the investigations of other physiologists, and especially because they show that the true cause of the changes induced by the division of the vagus nerves, is to be sought in the diminished quantity of air admitted in a given time into the lungs: thus, it matters not whether this diminution depends on the decreased number of respirations, as occurs when both vagi are cut; or on the paralysis of the glottis which mechanically impedes the entrance of the air, and which is induced, especially in young animals, and in all animals where the glottis is proportionally small, by the section indifferently of the vagi or recurrents; or on the obstruction produced by introducing solid bodies or liquids into the windpipe; in all these cases the diminished amount of oxygen introduced leads to an embarrassment of the pulmonary circulation, and to consequent effusion into the air-cells and small bronchial tubes. The fact is, that, in all the above instances, there is an approach to that condition, which in asphyxia attains its climax, and which consists in an obstruction situated in the rich vascular plexus spread over the walls of the air-cells, consequent, as we believe, on the absence of oxygen.

The explanation of Dr. Mendelssohn is, however, different to that just given; the principal object of his work is indeed to prove that, during the normal expansion of the lungs, the pulmonary artery, together with its branches and the capillaries proceeding from them, are all elongated, and thus attract the blood from the right side of the heart by suction; and that it is the loss of this power which leads to the congestion and effusion. The following extract will suffice to convey the author's opinion upon this point. "If an opening be made into the pleura, and a communication with the external atmosphere be thus caused, the lung immediately collapses and no longer expands during inspiration. The lengthening of the vessels, in virtue of which the blood is sucked into them out of the right ventricle, also ceases; and thus, one of the causes accomplishing the circulation in the lungs failing, the blood must move more slowly and with difficulty. The want of the power exerted by expiration also contributes to the same result; for, in the normal process, the air contained in the lungs, is, by the force of expiration, so much compressed, as to acquire a density exceeding that of the atmosphere, and in this manner it exerts a general pressure upon the pulmonic capillaries and tends to empty them of their contents. The blood in the collapsed lung is propelled only by a power which is equal to a part of the contractile force of the right ventricle, the pressure, which normally is exercised by means of the motion of the lung and thereby promotes the pulmonary circulation, being entirely absent." After stating that the vessels are in this condition pumped full of blood by the right ventricle, the author observes, "the dilatation of the capillaries and the exudation of a large portion of the plasma into the areolar tissue of the lungs and into the air-cells are the necessary consequence of the circumstances above described."—(p. 142.) This theory is similar to the views supported by the older physiologists, that in asphyxia the collapse of the lungs is the essential cause of that impediment to the pulmonary circulation which it is well known occurs, but which, in the present day, is attributed to the cause we have assigned, the want, namely, of oxygen, an explanation that is not likely to be shaken by the experiments and arguments adduced by Dr. Mendelssohn.

In the second part of the work, occupying two hundred pages, the author enters into a long and somewhat tedious discussion respecting the several varieties of pneumonia, seeking to apply his physiological researches to pathology; but as a consideration of this branch of the inquiry would necessarily exceed our limits, we must here conclude by remarking that, although these researches are unnecessarily overlaid by a large number of extracts from various writers, and present little that is original, they may be useful to the physiologist by confirming the results obtained from other sources.

